# Effect of storage medium of extracted human teeth on the result of in vitro studies – a scoping review

**DOI:** 10.2340/biid.v12.44183

**Published:** 2025-07-22

**Authors:** Muhammad Sadiq Billoo, Muhammad Ahsan Kaleem Khan, Taimur Khalid, Syed Murtaza Raza Kazmi

**Affiliations:** Department of Dentistry and Oral Health Sciences, The Aga Khan University, Karachi, Pakistan

**Keywords:** Storage media, composite, resins, microleakage, bond strength, micromechanical properties

## Abstract

**Purpose:**

This scoping review aimed to investigate how different storage media of extracted human teeth before and after preparation of specimens influence the outcomes of various dental materials intended for clinical application.

**Materials and methods:**

Literature search in four databases and by manual searching was carried out on a predefined PIO as follows: **P**opulation – extracted human teeth; **I**ntervention – different storage media; **O**utcome(s) – material properties.

**Results:**

After screening 110 studies, 11 were included in the scoping review. Eight studies examined storage media effects before specimen preparation and three after specimen preparation. Four studies focused on enamel bonding, six on dentine, and one both on enamel and dentine. Concerning storage of extracted teeth prior to specimen preparation, cryopreservation, Chloramine T and thymol had no negative effect on bond strength to enamel. One study found Chloramine T to increase microleakage at enamel margins whereas another study found no negative effect. Storage in formalin, ethanol and thymol also had no negative effect on microleakage at enamel margins. Cryopreservation decreased bond strength to coronal dentine, but not to mid-coronal or deep dentine. Formalin, Chloramine T, ethanol and thymol had no negative effect on bond strength to dentine nor on microleakage at dentine margins. However, one study found long-term storage in Chloramine T to increase microleakage at dentine margins. Concerning storage of extracted teeth after specimen preparation, thymol significantly lowered bond strength to enamel, whereas formalin had no negative effect. Sodium hypochlorite had no negative effect on bond strength to dentine, and finally, formalin, Chloramine T, ethanol, and thymol had no negative effect on microleakage at dentine margins. Risk of bias assessment showed one high quality study, while the remaining 10 had questionable quality.

**Conclusions:**

The effects of storage media on in-vitro dental material testing are variable and substrate-dependent, with dentine showing greater susceptibility than enamel. While distilled water and cryopreservation showed relative stability, other media like thymol and Chloramine T produced inconsistent results. Standardized protocols and further research are needed to ensure reliable and comparable outcomes.

## Introduction

Dental materials are continuously developed by scientists and researchers for patient care [1]. Before clinical utilization, these materials must undergo in-vitro testing [2] followed by their evaluation in pre-clinical trials for in-vivo application [3]. Extracted human teeth commonly serve as pivotal substrates for in-vitro testing of dental materials including dental restorative materials, effectively simulating the oral environment [4]. Although, alternatives such as teeth from shark [5], bovine [6, 7], primate [8], equine [8, 9] and porcine [8, 10] are available and match the properties of human dental tissues [11, 12], bovine teeth have been utilized more commonly in dental research due to their availability [12]. However, bovine teeth exhibit a faster rate of demineralization as their enamel is more porous than that of a human tooth [13–16]. Therefore, despite the convenience of using bovine teeth, human teeth remain indispensable in dental research [17]. They are crucial not only because of their easy availability but also for hands-on training in dental education as they provide a real time simulation to the teeth found in human clinical settings, providing a more relevant practice experience [3].

Challenges persist with human extracted teeth, including the risk of potential cross infection and the need for proper storage to preserve their properties till the time of their usage [18]. Suitable media required for their sterilization [4, 18–20] and storage are often inaccessible to dental researchers and practitioners in some parts of the world [21]. This storage of teeth before testing of any material properties is termed as ‘pre-test storage’ [11]. It is important to prevent post-extraction dehydration which greatly alters the mechanical properties of teeth [22]. A spectrum of storage media exists in literature and practice occurring in different states and forms, such as distilled water [23, 24], sodium hypochlorite (NaOCl) [25], Chloramine T [2, 14, 26], 4% formalin [23, 27], Hank’s Balanced Salt Solution (HBSS) [28], freezing at -20°C [29] and cryopreservation at -196°C [14, 30, 31]. Freezing at -20°C has been cited by some as the most conservative storage method, preserving the properties of dental substrates in a condition comparable to freshly extracted teeth. In contrast, cryopreservation involves storing specimens, including human teeth, at extremely low temperatures for extended periods. This method has been shown to effectively maintain dentine permeability and minimize microleakage without compromising the substrate [14, 32, 33]. However, comparative evaluations have not been explored in the literature to date with regards to the maintenance of properties of materials tested on extracted teeth. Therefore, a consensus on the optimal storage medium for maintaining the properties of the tested materials remains elusive [34]. Understanding the impact of different storage media on the results of tests is essential, as these variations may significantly alter the outcomes, potentially influencing the properties of the restorative materials being evaluated [1].

The lack of systematic literature support and robust evidence necessitates a thorough exploration of storage methods for extracted teeth. Guiding researchers on utilizing storage media effectively is crucial, emphasizing the current gap that requires addressing to identify a reliable and proven method for storing extracted teeth in preparation for in-vitro testing. The aim of this scoping review was to assess the impact of different storage media of extracted human teeth before and after preparation of specimens on the outcomes of in vitro tests of dental materials, with the goal of identifying the most effective method for preserving the properties of the teeth during in-vitro testing.

## Materials and methods

**Figure 1:**
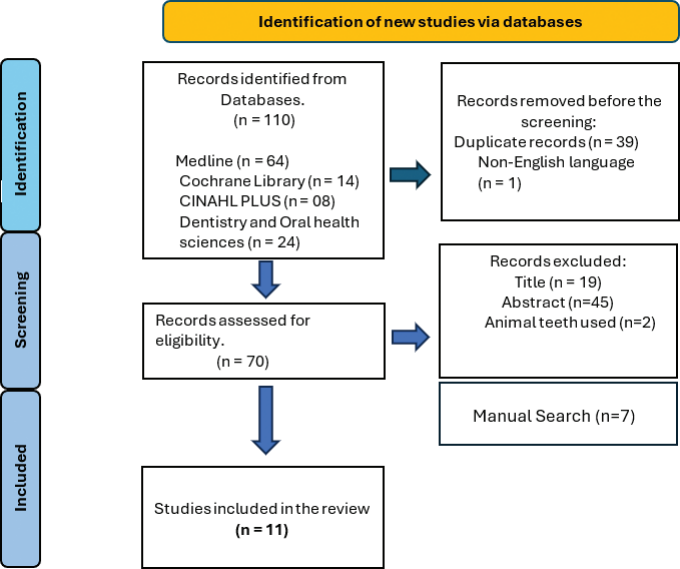
Prisma guidelines.

This scoping review adhered to the PRISMA Extension for Scoping Reviews guidelines [35] (Figure 1) and utilized the PIO framework: P – Extracted human teeth, I – Various storage media, O – Material properties. The electronic search conducted in September 2024 covered the following databases: Medline/PubMed, EBSCO CINAHL, EBSCO Dentistry and Oral Health Sciences, and Cochrane. The following search strategy was employed: (‘Composite Resins’[Mesh] OR Resin[tiab]) AND (‘Tooth Extraction’[Mesh: NoExp] OR ‘extracted teeth’ OR ‘extracted tooth’) AND (‘Storage Medi*’ OR storage OR water OR ‘physiologic saline’). Manual searches supplemented database findings to ensure inclusion of relevant studies. Registration was completed via Open Science Framework (OSF) at https://doi.org/10.17605/OSF.IO/JTDR8 [36].

### Selection criteria

Inclusion criteria involved in-vitro experimental studies featuring pre-test storage of permanent, extracted human teeth and subsequent dental material testing. Exclusion criteria encompassed deciduous human teeth, animal teeth studies, literature reviews, case reports, studies involving aging, and non-English language publications.

### Data extraction

Data extraction was conducted by two reviewers (MSB and MAK) through independent screening, removing duplicates and shortlisting studies based on title, objectives, and abstracts. Discrepancies were resolved through discussion and consensus with MTK. A calibrated proforma, validated by SMRK and MTK, was employed to systematically review selected articles.

### Risk of bias analysis

Risk of bias was assessed by Quality Assessment Tool for In-vitro Studies (QUIN) [37], where two reviewers (MSB and MAK) independently evaluated each study based on predefined criteria and formula.

Each item was graded as being of ‘low risk’, ‘medium risk’ and ‘high risk’ according to QUIN criteria. Each criterion was graded as: ‘2’ if it was clearly met, ‘1’ if there was ambiguity and ‘0’ if not clearly met. All criteria were summed up and divided from a total of 12 and the score was calculated. If the score was less than 50, the risk of bias was classified as ‘high risk’, if the score was between 50 and 70 the risk was ‘medium’, whereas a score higher than 70 meant the risk of bias was ‘low’ (Table 1).

**Table 1:** QUIN tool.

First Author, Year	1. Clearly stated objectives	2. Sample size calculation	3. Explanation of sampling technique	4. Comparison group	5. Method-ology	6. Operator details	7. Random-ization	8. Outcome measure-ment	9. Outcome assessor details	10. Blinding	11. Statistical analysis	12. Present-ation of results	Risk of Bias*
Retief et al., 1989 [40]	2	0	0	2	2	0	0	2	0	0	2	2	Medium
Panighi et al, 1997 [31]	2	2	1	2	2	0	0	2	0	0	2	2	Medium
Buoroziniat et al., 2017 [2]	2	1	0	2	2	0	0	2	0	0	2	2	Medium
De Roo et al., 2020 [30]	2	2	2	2	2	0	1	2	0	0	2	2	Low
Silva et al., 2015 [25]	2	0	0	2	2	0	0	2	0	0	2	2	Medium
Tosun et al., 2007 [23]	2	0	0	2	2	0	0	2	0	0	2	2	Medium
Mobarak et al., 2010 [39]	2	0	1	2	2	0	0	2	0	0	2	2	Medium
Toledano et al., 2007 [24]	2	0	0	2	2	0	0	2	0	0	2	2	Medium
Branstorm et al., 1992 [27]	2	0	0	2	2	0	0	2	0	0	2	2	Medium
Camps et al.,1996 [14]	2	0	1	2	2	0	1	2	2	0	2	2	Medium
Haller et al., 1993[27]	2	0	0	2	2	0	0	2	2	0	2	2	Medium

* Score: Adequately specified = 2; inadequately specified = 1; Not specified (NS) = 0; not applicable (NA); Final score; total score x100/2 x number of criteria applicable. >70% = low risk of bias; 50–70% = medium risk of bias; and < 50% = high risk of bias.

## Results

An initial electronic search identified 110 studies. After removing 39 duplicate records and excluding one non-English study, 70 studies remained for screening. Title screening led to the exclusion of 19 records, followed by the removal of 44 additional records after abstract review. Furthermore, two studies that utilized animal teeth were excluded, leaving five studies. One additional study by De Souza et al. [38] was further excluded after a detailed review, as it focused solely on the effects of long-term water storage only, leaving four studies. Additionally, seven studies identified through manual search were included, resulting in a total of 11 studies for the scoping review (Figure 1).

### Study characteristics

The 11 studies are listed in Table 2. Eight studies evaluated the effect of storage medium before specimen preparation [2, 14, 26, 27, 30, 31, 39, 40], while three studies examined after preparation [23–25]. Regarding tissue type, four studies examined the effect on enamel [2, 23, 26, 30], six studies examined the effect on dentine [14, 24, 25, 31, 39, 40], while one study examined the effect on both substrates [27]. With respect to the type of in-vitro test, three studies examined the effect of storage medium on shear bond strength (SBS) [30, 31, 40], three studies measured microshear bond strength (MSBS) [2, 23, 25], two studies measured microtensile bond strength (MTBS) [24, 39], while the last three studies measured microleakage [14, 26, 27].

**Table 2:** Characterization (storage of teeth before or after specimen preparation, substrate studied, test applied) and major findings of the 11 studies included.

Study	Timing	Substrate	Test	Findings
De Roo et al. [30]	Before	Enamel	SBS	Cryopreservation resulted in SBS values similar to storage in physiological saline (*p* > 0.05).
Bouroziniat et al. [2]	Before	Enamel	MSBS	No significant difference was observed amongst storage groups (freshly extracted teeth, 0.5% Chloramine T, 0.5% Chloramine T + autoclaving, 0.04% thymol, or 0.04% thymol + autoclaving) (*p >* 0.05). MSBS values after 6 months storage were significantly higher than after 12-month storage (*p* = 0.014).
Brännström et al. [26]	Before	Enamel	Microleakage	Storage in Chloramine T led to significantly more microleakage (wider gaps) compared to storage in frozen medium (*p* = 0.0004).
Panighi et al. [31]	Before	Dentine	SBS	Compared to freshly extracted teeth, cryopreservation resulted in lower SBS to coronal dentine (*p* = 0.013), but not to mid-/deep coronal dentine (*p* = 0.052).
Retief et al. [40]	Before	Dentine	SBS	Compared to storage in physiological saline, neither storage in buffered formalin, 1% Chloramine, 70% ethanol nor 0.05% thymol had any significant effect on SBS values (*p >* 0.05).
Mobarak et al. [39]	Before	Dentine	MTBS	No significant difference was observed amongst storage groups: freshly extracted teeth, 0.5% Chloramine T, or dry storage at room temperature (*p* > 0.05).
Camps et al. [14]	Before	Dentine	Microleakage	Compared to freshly extracted teeth, cryopreservation and short-term refrigeration in 0.5% Chloramine T had no effect on microleakage, whereas long-term refrigeration (≥48 d) resulted in increased microleakage (*p* = 0.001).
Haller et al. [27]	Before	Enamel & dentine	Microleakage	No significant difference was seen in microleakage amongst test (Formalin, 1% Chloramine, ethanol and thymol) and control groups (freshly extracted teeth) (*p <* 0.05).
Tosun et al. [23]	After	Enamel	MSBS	Compared to storage in distilled water, storage in thymol resulted in lower MSBS (*p* < 0.05), while storage in formalin resulted in MSBS values similar to storage in distilled water (*p >* 0.05). There were no statistically significant differences between the two storage periods for each solution (*p* > 0.05).
Da Silva et al. [25]	After	Dentine	MSBS	Storage in 1% NaOCl resulted in MSBS similar to storage in mineral oil or distilled water (*p* = 0.075). MSBS values were higher after 24 h storage than after 15 d (*p* < 0.005).
Toledano et al. [24]	After	Dentine	MTBS	Storage in water resulted in lower MTBS values as compared to dry and mineral oil storage (*p* < 0.0001).

* *p* ≤ 0.05 = significant, MSBS: microshear bond strength; SBS: shear bond strength; MTBS: microtensile bond strength; NaOCl: sodium hypochlorite.

### The effect of storage medium on teeth before specimen preparation

Of the eight studies evaluating storage before specimen preparation, six found no significant differences in test outcomes compared to freshly extracted controls [2, 14, 27, 30, 39, 40]. One study identified negative effects of certain storage conditions [26], while one study found a negative effect depending on the dentine level tested [31].

For enamel, one study found that cryopreservation resulted in SBS values statistically similar to storage in physiological saline (*p* > 0.05) [30]. Similarly, Borouziniat et al. reported no significant difference in MSBS to enamel amongst the different test groups (autoclaving, 0.5% Chloramine T and 0.04% thymol) and the control group (freshly extracted teeth) [2]. Whereas Haller et al. [26] found no negative effect of storage in 1% Chloramine T, formalin, ethanol or thymol on microleakage at enamel margins, by reporting statistically non-significant differences amongst the test groups (*p >* 0.05), Brännström et al. found significantly more microleakage in enamel when teeth were stored in 1% Chloramine T than in teeth that had been frozen [26].

For dentine, one study reported that cryopreservation led to lower SBS in coronal dentine, when compared to freshly extracted teeth but not in mid-coronal and deep-coronal dentine [31]. Another study found no significant effect of storage in physio-logical saline, buffered formalin, 1% chloramine, 70% ethanol or 0.05% thymol on SBS values (*p >* 0.05) [40]. Similarly, Mobarak et al. reported no statistically significant difference in MTBS following dry storage or storage in 0.5% Chloramine T compared to freshly extracted teeth (*p* > 0.05) [39]. Microleakage in dentine remained unchanged in cryopreserved specimens and short-time refrigerated specimens stored in 0.5% Chloramine T compared to freshly extracted teeth [14]. However, long-term storage in refrigerated 0.5% Chloramine led to a significant increase in microleakage in dentine (*p* = 0.001) [14] Finally, Haller et al. reported no negative effect of storage in 1% Chloramine T, formalin, ethanol or thymol on microleakage at dentine margins [27].

### The effect of storage medium on teeth after specimen preparation

Among the three latter studies examining post-preparation effects, one found no negative effect [25], one study [23] found a negative effect of one medium but not of the other, and finally one study [24] found a negative effect of storage in water compared to mineral oil and dry storage.

For enamel, Tosun et al. reported significantly lower MSBS values (*p* < 0.05) in specimens stored in thymol compared to formalin and distilled water, and they observed no significant differences in MSBS values within each group over time [23].

For dentine, Da Silva et al. observed statistically similar bond strength of specimens stored in 1% NaOCl, mineral oil and distilled water (*p* = 0.075), although prolonged storage in test media decreased MSBS significantly (*p* < 0.005) [25]. In contrast, Toledano et al. found that MTBS significantly decreased following water storage compared to dry and mineral oil storage (*p* < 0.0001) [24].

### Risk of bias assessment

Risk of bias assessment using the QUIN tool identified only one study with low risk of bias and 10 studies with medium to high risk of bias (Table 1). Only one study provided detailed sample size calculations and outcome assessor information. None of these studies reported randomization, and operator details were generally missing.

## Discussion

This scoping review investigated the influence of tooth storage media on in vitro testing outcomes in dental materials research, addressing previously inconsistent and conflicting evidence. Given the variability in methodologies and findings across the literature, the aim was to synthesize current evidence and identify trends, limitations, and future directions.

### Impact of storage media before versus after specimen preparation

The review findings suggest that the timing of storage, whether before or after specimen preparation, can significantly influence test outcomes. Mobarak et al. [39]. found no significant differences in MTBS between freshly extracted teeth and those stored dry or in Chloramine T, prior to specimen preparation. This supports the suitability of these media for short-term preservation. In contrast, Brannstrom et al. [26] observed increased enamel microleakage in specimens stored in Chloramine T versus those frozen before preparation, indicating that certain storage conditions may compromise structural integrity.

Studies evaluating post-preparation storage, such as De Souza et al. [38], emphasized that prolonged water storage can lead to hydrolytic degradation and increased microleakage. Variability in bond strength outcomes following thymol storage, reported by Boruziniat et al. [2] and Tosun et al. [23] may also be attributed to differences in the timing and conditions of storage.

These results collectively demonstrate that the effect of storage media is influenced not only by the medium itself but also by the stage at which it is applied. Absorption of Chloramine T into dentinal tubules, previously noted by Rolland et al. [41], may further alter the substrate’s bonding behavior.

### Differential effects on enamel versus dentine

Further analysis revealed that enamel and dentine may respond differently to storage media, likely due to intrinsic structural and compositional differences [42]. Among the five studies that included enamel, three found no adverse effect of storage media compared to the control, while one reported reduced MSBS values [23] and another identified a negative effect from one medium, but not another [26]. The decreased MSBS values in enamel may stem from increased porosity following specimen preparation [43], which can enhance fluid absorption and alter the tissue’s response to storage conditions.

In the seven studies that evaluated dentine, four reported no significant impact of storage medium, two observed negative effects, one implicating water storage [23] and one showed dentine depth dependent variation [31]. The increased microleakage observed by Camps et al. [14] may be explained by dentine’s higher permeability and tubule density, which increase its susceptibility to fluid-based degradation [44]. These findings suggest that dentine may be more vulnerable to storage-induced changes than enamel, though no consistent tissue-specific differences were observed across all studies. Nevertheless, due to dentine’s greater heterogeneity and biological sensitivity, it remains a more critical substrate for avoiding material testing artifacts, especially with water-based storage.

### Impact of storage media based on type of in vitro test

The review also identified heterogeneity in testing methodologies, with eight studies evaluating bond strength and three focusing on microleakage.

In SBS studies, cryopreservation was associated with slightly reduced SBS values compared to fresh specimens, as noted in coronal dentine by Panighi et al. [31] and in enamel by De Roo et al. [30] Although these differences were minor, these findings warrant cautious interpretation and suggest large-scale controlled trials.

The most frequently studied storage media were Chloramine T and thymol, though with mixed results. Thymol was consistently associated with reduced SBS, as demonstrated by Retief et al. [40], aligning with decreased MSBS values in other studies. While Boruziniat et al. [2] found no significant difference between thymol, Chloramine T and freshly extracted teeth, Secilmis et al. [43] reported that thymol altered dentine mineral content, possibly affecting bonding. Additionally, Fujisawa and Kadoma et al. [44] identified that thymol reacts with free radicals, interfering with polymerization of methyl methacrylate. Tosun et al. [23] found lower MSBS in thymol-stored teeth compared to those stored in distilled water and formalin, discouraging its use for bond strength testing. Conversely, distilled water emerged as a reliable storage medium in studies by Tosun et al. and De Silva et al., while Boruziniat supported the neutrality of Chloramine T in maintaining bond strength during pre-test storage.

In microleakage studies, prolonged Chloramine T exposure was linked to increased leakage due to adhesive interface degradation as shown by Brannstrom et al. [26] and Camps et al. [14] However, Haller et al. [27] reported more favorable outcomes with Chloramine T, suggesting variability depending on storage duration and test conditions [45].

Formalin was generally discouraged due to its effect on collagen cross-linking, which may alter the mechanical properties of dental substrate and yield results that differ from clinical behavior [46, 47]. Additionally, Camps et al. [14] observed increased microleakage in prolonged freezing due to void formation in the resin layer. Despite this, cryopreservation appeared otherwise effective, with minimal impact over storage periods of up to 18 months, supporting its use for long-term preservation [14].

MTBS was evaluated in only two studies [24, 39] likely due to the technical demands of producing small specimens with uniform surface area. [45] Despite the complexity, MTBS is considered more reliable as it distributes stresses uniformly across the adhesive interface and enables multiple tests per tooth [48]. Toledano et al. [24] reported decreased MTBS values with water storage, possibly due to the activation of matrix metalloproteinase (MMPs) enzymes that require water to hydrolyze collagen peptide bonds [49, 50]. While Mobarak et al. [39] supported Chloramine T’s suitability, the withdrawal of ‘ISO/TS 11405:2003 and ISO/TS 11405:2015’ [51] underscores the need for revised standards and further research.

### Least utilized storage media and future directions

Storage media such as physiological normal saline, phosphate buffered saline and freezing were among the least utilized storage media (Table 3), yet they warrant further investigations through well-designed controlled trials. The current evidence indicates that dry storage, formalin, and cryopreservation had the least negative impact on testing outcomes, though each has limitations. For example, while dry storage may reduce microbial contamination, it can also lead to dentine desiccation and lower SBS as observed in bovine teeth [15]. Similarly, while some studies suggest formalin is chemically neutral, its effect on collagen structure may lead to artificially altered results. Notably, formalin is typically considered safe for sterilization only in 10% concentration for up to 1 week.

**Table 3:**
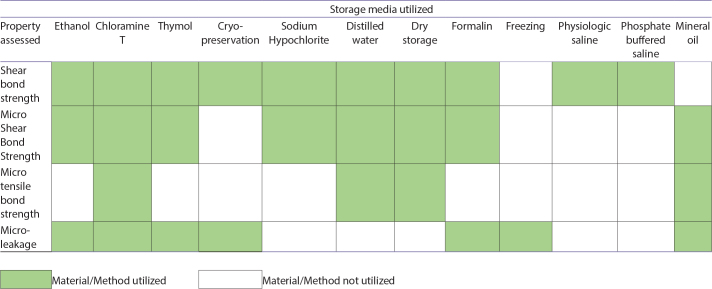
Storage media utilized, and properties assessed.

Cryopreservation, though technically demanding, has shown promise as a storage method for indefinite preservation, pending further validation in human specimens [52].

### Risk of bias and study limitations

A high or unclear risk of bias was identified in most of the included studies (*n* = 10), largely due to missing details regarding operator calibration, randomization procedures, and outcome assessor blinding. This review used the QUIN tool for bias assessment. However, future reviews could benefit from more specialized tools such as RoBDEMAT, which is tailored to dental materials research [53].

This review’s strengths include a systematic and structured risk-of-bias assessment and its novelty as the first scoping review to focus specifically on the impact of storage media on human extracted teeth used in dental materials testing. However, several limitations must be acknowledged, including the heterogeneity of methodologies, the absence of meta-analytic synthesis, and the limited number of recent controlled trials. These issues highlight the urgent need for standardized storage protocols and further high-quality research to validate findings and ensure reproducibility in in-vitro dental studies.

## Conclusion

This scoping review highlights the critical impact of storage media on in-vitro testing outcomes utilizing extracted human teeth. While Chloramine T and thymol demonstrated variable influence depending on the substrate, storage duration and test type, distilled water and cryopreservation appear more stable under specific conditions. The differential responses between enamel and dentine, particularly the heightened susceptibility of dentine due to its structural properties, underscore the importance of substrate-specific considerations. Despite inconsistencies, no universally optimal storage medium was identified. Methodological differences and potential biases limit current evidence, emphasizing the need for standardized protocols and rigorous studies to improve reproducibility in dental research, particularly in lab testing of dental materials.
